# Effects of temporal changes in resting heart rate on future diabetes-related outcomes

**DOI:** 10.3389/fendo.2024.1385143

**Published:** 2024-04-18

**Authors:** Lu Gao, Guo-Hong Wang, Gang Wan, Qian Liu, Ming-zhao Qin, Fang Fang, Xue-li Cui, Yu-ling Li, Fei Sun, Xue-lian Zhang, Han-jing Fu, Shen-yuan Yuan

**Affiliations:** ^1^ Department of Geriatrics, Beijing Tongren Hospital, Capital Medical University, Beijing, China; ^2^ Department of Medical Records and Statistics, Beijing Ditan Hospital, Capital Medical University, Beijing, China; ^3^ Sanlitun Community Health Service Center, Beijing, China; ^4^ Xinjiekou Community Health Service Center, Beijing, China; ^5^ Cuigezhuang Community Health Service Center, Beijing, China; ^6^ Department of Endocrinology, Beijing Tongren Hospital, Capital Medical University, Beijing, China

**Keywords:** resting heart rate, change, long-term, clinical outcomes, type 2 diabetes mellitus

## Abstract

**Background and aims:**

Most studies have analyzed the relationship between resting heart rate (RHR) measured at only one time point and future clinical events. The current study aims to investigate the impact of long-term RHR changes on future clinical outcomes in a decade-long cohort with type 2 diabetes mellitus (T2DM).

**Methods:**

The two-staged follow-up involved 2,513 T2DM participants. The first stage (2008-2014) intended to identify levels and trends in RHR changes, while the second stage (2014-2018) attempted to collect new occurrence records of clinical results. Cox proportional hazards models were applied to predict hazard ratios (HRs), along with 95% confidence interval (CI) for the correlation between RHR changes and future events.

**Results:**

There is no significant correlation between baseline RHR levels and long-term clinical events. According to the range of RHR change, compared with the stable RHR group, the adjusted HRs for cardiovascular events and all-cause death in the large increase group were 3.40 (95% CI: 1.33-8.71, p=0.010) and 3.22 (95% CI: 1.07-9.64, p=0.037), respectively. While the adjusted HRs for all-cause death and major adverse cardiac and cerebrovascular events (MACCE) in the moderate decrease group were 0.55 (95% CI: 0.31-0.96, p=0.037) and 0.51 (95% CI: 0.26-0.98, p=0.046). According to the trend of RHR, compared with the normal-normal group, the adjusted HRs for composite endpoint events and cerebrovascular events in the normal-high group were 1.64 (95% CI: 1.00-2.68, p=0.047) and 2.82 (95% CI: 1.03-7.76, p=0.043), respectively.

**Conclusion:**

Changes in RHR had predictive value for long-term clinical events in diabetic populations. Individuals with significantly elevated RHR over a particular period of time showed an increased risk of adverse events.

## Introduction

Individuals with type 2 diabetes (T2DM) form a large and ever-increasing population, whose risk of cardiovascular events and mortality has been rising rapidly. Studies have indicated that diabetes makes it twice as likely for the risk of mortality and an array of vascular diseases compared with non-diabetic individuals (1). T2DM patients may constitute a significant part of the overall burden of cardiovascular disease (CVD). It is therefore a key issue to identify before timely intervening the risk factors of complications of diabetes.

Resting heart rate (RHR) is a non-invasively measured marker of cardiac function and a strong and positive sign of general health (2). Elevated RHR has been confirmed to have an association with increased cardiovascular events and mortality. Such a link has been well-documented in apparently healthy people (3) and other individuals found in disease-specific populations, including hypertension (4), coronary heart disease (5, 6) and heart failure (7). Most of the current data are based on research on the correlation between RHR gauged at a particular time slot and subsequent CVD events. However, RHR does not remain stable throughout a lifetime; rather, it is a variable that changes in reaction to the interplay of genes and environmental causes, physical activity, clinical conditions and medical therapies.

For this reason, whether temporal changes in RHR have predictive value for long-term future events has stood out as an intriguing and crucial issue. Having said that, research on the relationship between long-term longitudinal trends of RHR and future clinical results is still far from abundant, not to mention research within the context of T2DM population. We thus aimed to categorize long-term RHR changes and evaluate their impact on future clinical outcomes in a cohort lasting 10 years, focusing on Chinese T2DM patients from a metropolitan city.

## Materials and methods

### Study population

The Beijing Community Diabetes Study (BCDS) is a prospective cohort study with participants from 15 community service centers in 5 local districts. To ensure consistent implementation of research processes across all health centers, all staff members underwent professional training, with frequent on-spot checks of clinical and laboratory examinations from experienced inspectors. A total of 4,256 T2DM patients (aged 20 to 80) who had lived locally for 5 years or longer were recruited for the study, being identified through screening of patient records. They were followed up annually for as long as 10 years (2008-2018), during which questionnaire surveys, physical examinations and biochemical indicators were all conducted, apart from electrocardiogram examinations. Considering that most clinical outcomes took place after 2014 (81%), we separated the decade-long follow-up into two stages for current data analysis to evaluate the impact of RHR and long-term RHR changes on future clinical outcomes. The first stage (2008-2014) intended to determine the patterns of RHR change, while the second stage (2014-2018) was designed to collect records of future clinical events related to diabetes.

**Figure 1:**
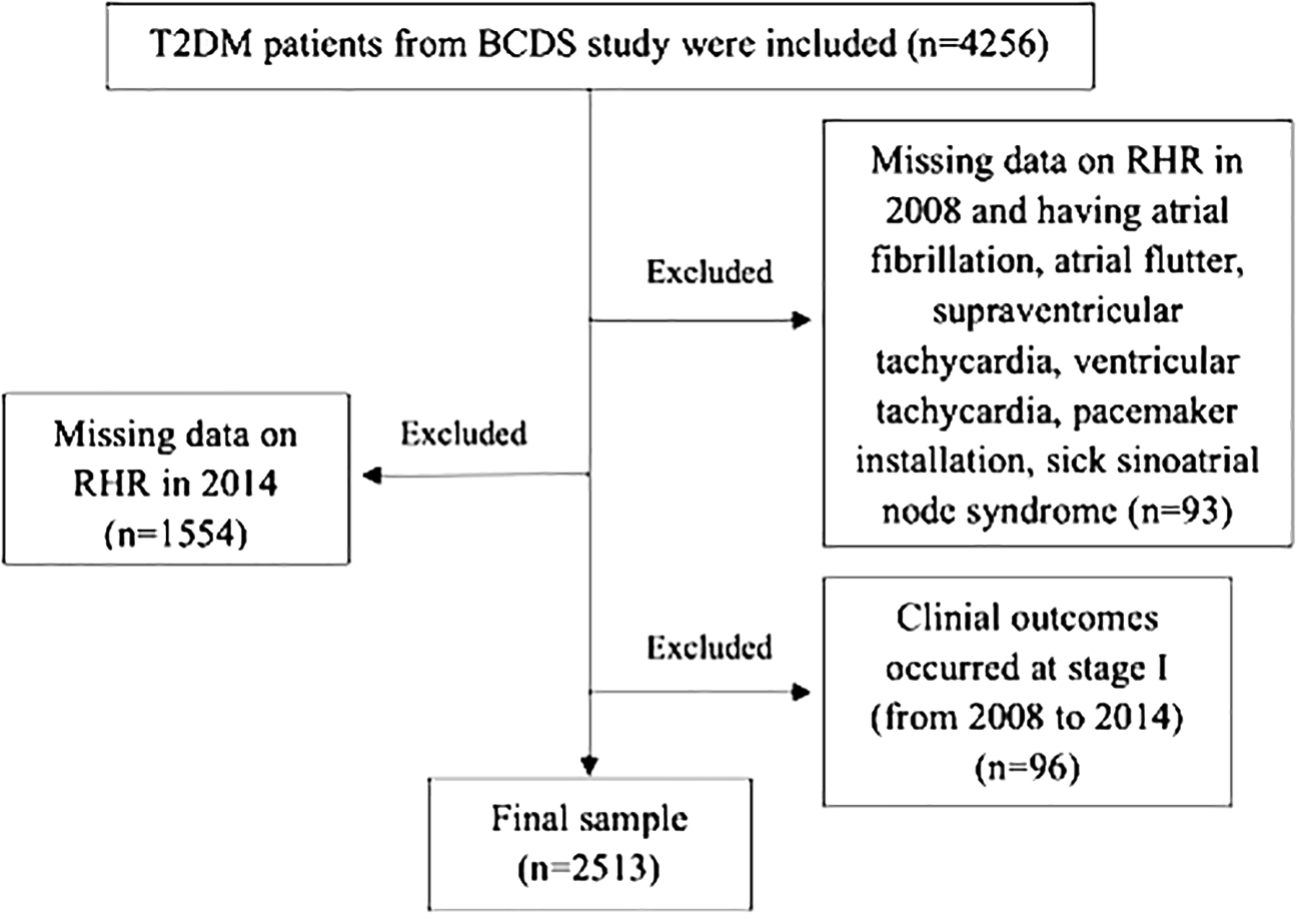
Flowchart of the BCDS study.

Participants with no RHR records and having a history of atrial fibrillation, atrial flutter, supraventricular tachycardia, ventricular tachycardia, pacemaker implantation and sick sinus syndrome during enrollment were excluded (n=93). Participants who did not have 2014 RHR records, missed outcome data or did not complete the entire follow-up were ruled out (n=1544). Those with clinical outcomes occurring in the first stage (2008-2014) were also excluded (n=96). Finally, 2,513 T2DM patients were included in the study (**Figure 1**).

### Ethics approval and consent to participate

The study was conducted with the approval from the Ethics Committee of Beijing Tongren Hospital, Capital Medical University. Written informed consent was obtained from each participant.

### Data collection

Each participant was given a standardized examination. Statistics in terms of age, smoking, level of education, concomitant disease and medication were acquired with detailed medical records. Body mass index (BMI) was calculated as weight (kg) divided by height squared (m^2^). Waist circumference (WC) was measured at the level of the umbilicus in cm. Blood pressure (BP) was measured 3 times when participants were seated, and the average of the last 2 measurements was adopted. Blood samples were collected after an overnight fast for the determination of plasma glucose, HbA1c, total cholesterol (TC), triglycerides (TG), high density lipoprotein cholesterol (HDL-C), low density lipoprotein cholesterol (LDL-C) concentrations and serum creatinine (Scr). GFR was estimated by using the modified MDRD formula for Chinese patients (8): eGFR (ml/min/1.73m^2^) = 175 × Scr^−1.234^ × age^−0.179^ × 0.79 (if female). All of the above were clinically measured by trained staff.

### Definition of clinical and biochemical variables

T2DM was defined according to the criteria of the WHO (1999) (9). The cutoff for diabetes is glucose level ≥7 mmol/l for fasting and ≥11.1 mmol/l for 2 hours (2h PG) or history of diabetes mellitus or taking antidiabetic medications. Diagnosis of hypertension was based on meeting any of the following criteria: systolic blood pressure (SBP) of ≥140 mmHg, diastolic blood pressure (DBP) of ≥90 mmHg or current use of antihypertensive drugs (10). Educational level was categorized into two groups: low (illiteracy, primary or secondary school) and high (high school, college or university). Smokers were defined as those who had smoked ≥1 cigarette/day for at least 12 months.

### Measurement of resting heart rate

After recumbent resting for 10 minutes in a quiet environment, all subjects underwent routine 12 lead electrocardiogram examination, followed by 10 cardiac cycles recorded in lead II. The average R-R interval was used to calculate RHR.

### Outcome ascertainment

Outcomes included all-cause death, cardiovascular events (acute myocardial infarction, coronary artery bypass grafting, coronary stent, unstable angina pectoris, hospitalization for heart failure and installation of cardiac pacemaker), cerebrovascular events (cerebral hemorrhage, cerebral infarction, transient ischemic attack and subarachnoid hemorrhage), major adverse cardiac and cerebrovascular events (MACCE) (recurrent myocardial infarction, new heart failure, intractable angina, cardiac death, stroke and cerebrovascular death), renal events (new-onset proteinuria, microalbuminuria turning into macroalbuminuria, doubling of the serum creatinine level and dialysis) and composite endpoint events (all-cause death, cardiovascular events, cerebrovascular events and renal events). All events occurred from 2014 to 2018. All outcomes are adjudicated by an independent committee responsible for verifying data and events based on outcome criteria.

### Statistical analysis

The baseline recorded RHR was categorized as <60 bpm, 60–69 bpm, 70–79 bpm, 80–89 bpm and >=90 bpm, consistent with previous publications (5, 11). Due to small sizes of the <60 bpm and >=90 bpm groups (23 and 13 subjects respectively), however, we merged <60 bpm group and 60–69 bpm group into <70 bpm group, as well as the >=90 bpm group and 80–89 bpm group into >=80 bpm group. Therefore, baseline RHR was categorized as three groups based on numerical magnitude: <70bpm, 70–79 bpm and >=80 bpm. In the second analysis, the associations between change in RHR (difference between the second recorded RHR and the baseline recorded RHR) and diabetes-related clinical events were assessed. With reference to the range of numerical change between the two recorded RHRs, temporal change of RHR can be divided into five groups: large decrease (over 15 bpm), moderate decrease (6-15 bpm), stable RHR (-5-+5bpm), moderate increase (6-15 bpm) and large increase (over 15 bpm), consistent with a previous publication (12). Participants with a stable RHR were used as the reference group. In addition, analyses were performed with RHR as a dichotomous variable, above and below 70 bpm as used in previous studies (11, 13), RHRs of 70 bpm or lower are considered normal, while RHRs greater than 70 bpm are considered high. According to the baseline and second recorded RHR trends, as used Participants were thus divided into four groups: normal-normal, normal-high, high-normal and high-high. For the discrete variables or the continuous variables without a normal distribution, the median (P25–P75) was reported. Comparison of variables among groups was performed using the Kruskal–Wallis and one-way ANOVA. In the meantime, distribution of discrete/qualitative variables was compared by Pearson chi-square test.

Cox proportional hazards models were used to estimate crude and adjusted hazard ratio (HR) with the 95% confidence interval (CI) to allow for differences between groups with respect to demographic and risk factors and control for potentially confounding variables. All potential confounders were included in the models, all measured at baseline: age, sex, the duration of diabetes, education level, smoking, waist circumference, concomitant disease history in baseline, ACEI/ARB use, β receptor blocker use, SBP, DBP, TC, HbA1c and eGFR.

Two-sided P-values were reported for all analyses. A p-value of less than 0.05 was considered statistically significant. All statistical analyses were conducted with SAS software (version 9.2; SAS Institute Inc., Cary, NC, USA).

## Results

### Demographic and biochemical parameters in the baseline

Demographic and biochemical parameters in the baseline are shown in **Table 1**. Statistical differences were registered from the three groups regarding clinical characteristics (age, levels of waist circumference, SBP, DBP, FPG, HbA1c, TC and eGFR, and the percentage of male, smoking, Metformin, ACEI and β receptor blockers use). The proportions of male and smoking in >=80 group were substantially higher than those in the other two groups, whereas the proportions of ACEI and β receptor blockers use were significantly lower. The levels of age and eGFR in >=80 group were considerably lower than those in <70 group and 70-79 group, while the levels of waist circumference, SBP, DBP, FPG, HbA1c and TC were significantly higher.

**Table 1:** Demographic and biochemical parameters of the three groups in the baseline.

	Total	Baseline RHR categories (bpm)			
		RHR <70	RHR 70-79	RHR >=80	P
**Number (%)**	2513	511	1485	517	
**Age(years)**	62.24 ± 10.27	63.32 ± 9.98	62.46 ± 10.11	60.51 ± 10.83	<0.001
**Male n(%)**	1003(39.91)	202(39.53)	573(38.59)	228(44.10)	0.086
**High educational level n(%)**	486(19.42)	100(19.76)	298(20.11)	88(17.12)	0.498
**Smoking n(%)**	372(14.82)	63(12.35)	206(13.89)	103(19.92)	0.001
**Duration of DM (years)**	12.21 ± 105.58	5.0(1.1,10.1)	4.7(0.9,9.9)	4.7(0.5,9.9)	0.636
**HT n(%)**	1824(72.58)	386(75.54)	1061(71.45)	377(72.92)	0.199
**History of cerebrovascular n(%)**	283(11.26)	63(12.33)	162(10.91)	58(11.22)	0.681
**History of cardiovascular n(%)**	488(19.42)	111(21.72)	283(19.06)	94(18.18)	0.307
**Metformin n(%)**	1145(45.56)	222(43.44)	679(45.72)	244(47.20)	0.473
**Sulfonylurea drugs n(%)**	614(24.43)	135(26.42)	343(23.10)	136(26.31)	0.173
**Insulin therapy n(%)**	540(21.49)	101(19.77)	326(21.95)	113(21.86)	0.568
**ACEI n(%)**	391(15.56)	93(18.20)	233(15.69)	65(12.57)	0.044
**ARB n(%)**	381(15.16)	92(18.00)	222(14.95)	67(12.96)	0.074
**Calcium antagonist n(%)**	974(38.76)	205(40.12)	585(39.39)	184(35.59)	0.242
**β receptor blockers n(%)**	302(12.02)	81(15.85)	168(11.31)	53(10.25)	0.009
**Diuretic n(%)**	92(3.66)	25(4.89)	55(3.70)	12(2.32)	0.089
**α receptor blockers n(%)**	17(0.68)	3(0.59)	12(0.81)	2(0.39)	0.672
**BMI (kg/m^2^)**	27.30 ± 68.91	25.32 ± 3.48	26.91 ± 63.32	30.37 ± 107.52	0.475
**WC (cm)**	88.77 ± 9.45	89.23 ± 9.76b	88.23 ± 9.37	89.86 ± 9.27	0.002
**SBP (mmHg)**	128.71 ± 13.45	127.07 ± 13.14	128.23 ± 12.59	131.66 ± 15.57	<0.001
**DBP (mmHg)**	77.53 ± 8.47	75.68 ± 9.01	77.41 ± 8.07	79.70 ± 8.58	<0.001
**FPG (mmol/l)**	7.70 ± 2.48	7.18 ± 2.11	7.68 ± 2.45	8.33 ± 2.78	<0.001
**PG 2h (mmol/l)**	11.02 ± 10.49	10.87 ± 12.16	10.97 ± 11.24	11.31 ± 4.57	0.793
**HbA1c (%)**	7.23 ± 1.49	7.02 ± 1.45	7.18 ± 1.45	7.58 ± 1.60	<0.001
**TG (mmol/l)**	1.89 ± 1.43	1.90 ± 1.44	1.84 ± 1.32	2.03 ± 1.72	0.052
**TC (mmol/l)**	5.15 ± 1.16	5.08 ± 1.18	5.14 ± 1.11	5.27 ± 1.26	0.031
**HDL-C (mmol/l)**	1.33 ± 0.46	1.30 ± 0.47	1.34 ± 0.47	1.32 ± 0.42	0.237
**LDL-C (mmol/l)**	3.01 ± 0.91	2.96 ± 0.88	3.01 ± 0.90	3.09 ± 0.93	0.113
**UAER**	11.1(7.2,23.6)	12.3(7.1,27.3)	10.5(7.2,21.6)	11.8(7.4,25.9)	0.271
**eGFR (ml/min)**	93.5(74.6,116.5)	95.8(74.3,118.5)	93.9(75.6,117.3)	89.4(72.3,111.8)	0.031

Data are means ± SE, median (P25–P75) or raw numbers (%). RHR, resting heart rate; DM, diabetes mellitus; HT, hypertension; ACEI, angiotensin-converting enzyme inhibitor; ARB, angiotensin receptor blocker; BMI, body mass index; WC, waist circumference; SBP, systolic blood pressure; DBP, diastolic blood pressure; FPG, fasting plasma glucose; 2h PG, 2-h post oral glucose load plasma glucose; HbA1c, glycated hemoglobin; TG, triglycerides; TC, total cholesterol; HDL-C, high- density lipoprotein; LDL-C, low-density lipoprotein cholesterol; UAER, urinary albumin excretion rates; eGFR, estimated glomerular filtration rate.

### Grouping according to the two patterns of RHR change and comparison of their baseline characteristics

With reference to the range of numerical change between the second recorded RHR and the baseline recorded RHR, there were statistical discrepancies among the five groups in the proportion of ACEI use and the levels of the duration of diabetes, SBP, DBP, FPG, HbA1c, UAER and eGFR (**Table 2**).

**Table 2:** Demographic and biochemical parameters of the groups according to the range of RHR change in the baseline.

	Change in RHR categories (bpm)					
	large decrease	moderate decrease	stable RHR	moderate increase	large increase	p
**Number**	83	511	1589	300	30	
**Age(years)**	62.58 ± 10.82	62.53 ± 10.69	61.95 ± 10.22	63.13 ± 9.66	62.53 ± 10.69	0.400
**Male n(%)**	29(34.94)	196(38.36)	647(40.72)	114(38.00)	17(56.67)	0.217
**High educational level n(%)**	11(13.41)	93(18.27)	325(20.50)	49(16.55)	8(26.67)	0.167
**Smoking n(%)**	14(16.87)	77(15.10)	232(14.61)	46(15.38)	3(10.00)	0.911
**Duration of DM (years)**	6.1(1.2,11.7)	5.1(1.5,11.1)	4.3(0.6,9.8)	4.9(1.3,9.8)	3.3(1.4,8.1)	0.016
**HT n(%)**	62(74.70)	367(71.82)	1143(71.93)	229(76.33)	23(76.67)	0.550
**History of cerebrovascular n(%)**	9(10.84)	64(12.52)	166(10.45)	39(13.00)	5(16.67)	0.453
**History of cardiovascular n(%)**	22(26.51)	106(20.74)	295(18.57)	59(19.67)	6(20.00)	0.407
**ACEI n(%)**	14(16.87)	78(15.26)	233(14.66)	56(18.67)	10(33.33)	0.032
**ARB n(%)**	5(6.02)	72(14.09)	250(15.73)	51(17.00)	3(10.00)	0.105
**Calcium antagonist n(%)**	28(33.73)	202(39.53)	618(38.89)	111(37.00)	15(50.00)	0.556
**β receptor blockers n(%)**	4(13.33)	51(17.00)	187(11.77)	53(10.37)	7(8.43)	0.051
**Diuretic n(%)**	1(1.20)	14(2.74)	59(3.71)	18(6.00)	0(0.00)	0.076
**α receptor blockers n(%)**	0(0.00)	3(0.59)	10(0.63)	4(1.33)	0(0.00)	0.630
**BMI (kg/m^2^)**	25.68 ± 3.41	25.24 ± 3.28	28.36 ± 86.62	25.76 ± 3.91	25.93 ± 3.57	0.904
**WC (cm)**	90.36 ± 9.05	88.25 ± 9.25	88.66 ± 9.50	89.66 ± 9.52	89.75 ± 10.46	0.134
**SBP (mmHg)**	131.77 ± 13.23a	130.65 ± 14.94a	127.82 ± 12.59a	128.97 ± 14.80a	131.20 ± 13.32a	<0.001
**DBP (mmHg)**	79.39 ± 8.43a	79.00 ± 8.39a	76.97 ± 8.22a	77.59 ± 9.24a	76.37 ± 11.20a	<0.001
**FPG (mmol/l)**	8.13 ± 2.63	8.03 ± 2.71	7.62 ± 2.42	7.37 ± 2.21	8.46 ± 2.90	<0.001
**PG 2h (mmol/l)**	11.25 ± 4.66	11.45 ± 10.70	11.00 ± 11.57	10.33 ± 3.88	10.75 ± 4.55	0.728
**HbA1c (%)**	7.38 ± 1.49a	7.43 ± 1.63a	7.15 ± 1.41a	7.18 ± 1.62a	7.40 ± 1.44a	0.007
**TG (mmol/l)**	1.90 ± 1.19	1.98 ± 1.55	1.86 ± 1.43	1.88 ± 1.37	1.80 ± 0.98	0.659
**TC (mmol/l)**	5.15 ± 1.05	5.22 ± 1.22	5.10 ± 1.13	5.23 ± 1.21	5.59 ± 1.27	0.051
**HDL-C (mmol/l)**	1.33 ± 0.37	1.34 ± 0.55	1.31 ± 0.42	1.37 ± 0.51	1.43 ± 0.71	0.222
**LDL-C (mmol/l)**	3.05 ± 0.83	3.07 ± 0.89	2.98 ± 0.90	3.05 ± 0.91	3.22 ± 1.43	0.289
**UAER**	10.9(7.1,18.6)	11.9(7.2,27.4)	10.6(7.1,21.6)	14.3(8.0,28.8)	7.1(6.2,14.4)	0.026
**eGFR (ml/min)**	99.6(72.8,122.4)	92.3(73.7,117.1)	94.7(76.4,116.6)	89.1(71.1,112.3)	82.2(67.9,105.7)	0.036

According to the baseline and second recorded RHR trends, statistically significant differences were seen with the four groups in the proportion of males, smoking and diuretic use, as well as the levels of age, SBP, DBP, FPG, HbA1c and TC (**Table 3**).

**Table 3:** Demographic and biochemical parameters of the groups according to the trend of RHR change in the baseline.

	Change in RHR categories (bpm)				
	normal-normal	normal−high	high−normal	high−high	P
**Number**	570	383	500	1060	
**Age(years)**	63.31 ± 9.69	63.14 ± 10.55	62.27 ± 10.40	61.32 ± 10.35	0.001
**Male n(%)**	203(35.61)	169(44.13)	195(39.00)	436(41.13)	0.045
**High educational level n(%)**	118(20.77)	74(19.58)	100(20.16)	194(18.30)	0.803
**Smoking n(%)**	57(10.02)	55(14.40)	74(14.83)	186(17.55)	0.001
**Duration of DM (years)**	4.9(1.1,10.0)	4.7(1.3,9.8)	5.0(1.1,11.1)	4.4(0.5,9.7)	0.188
**HT n(%)**	419(73.51)	284(74.15)	357(71.40)	764(72.08)	0.751
**History of cerebrovascular n(%)**	66(11.58)	47(12.27)	68(13.60)	102(9.62)	0.110
**History of cardiovascular n(%)**	117(20.53)	68(17.75)	96(19.20)	207(19.53)	0.765
**ACEI n(%)**	86(15.09)	67(17.49)	88(17.60)	150(14.15)	0.224
**ARB n(%)**	101(17.72)	60(15.67)	71(14.20)	149(14.06)	0.227
**Calcium antagonist n(%)**	234(41.05)	155(40.47)	196(39.20)	389(36.70)	0.299
**β receptor blockers n(%)**	79(13.86)	54(14.10)	58(11.60)	111(10.47)	0.118
**Diuretic n(%)**	24(4.21)	24(6.27)	14(2.80)	30(2.83)	0.012
**α receptor blockers n(%)**	2(0.35)	4(1.04)	4(0.80)	7(0.66)	0.568
**BMI (kg/m^2^)**	29.30 ± 102.08	25.65 ± 3.76	25.33 ± 3.49	27.75 ± 75.13	0.764
**WC (cm)**	88.33 ± 9.44	89.67 ± 8.95	88.33 ± 9.51	88.88 ± 9.60	0.118
**SBP (mmHg)**	126.48 ± 12.74b	127.14 ± 13.03b	129.34 ± 13.49a	130.17 ± 13.75a	<0.001
**DBP (mmHg)**	75.90 ± 8.52b	76.35 ± 8.98b	78.06 ± 7.88a	78.58 ± 8.35a	<0.001
**FPG (mmol/l)**	7.30 ± 2.23	7.40 ± 2.23	7.85 ± 2.60	7.97 ± 2.60	<0.001
**PG 2h (mmol/l)**	11.93 ± 18.17	10.38 ± 3.90	10.67 ± 6.87	10.93 ± 7.35	0.122
**HbA1c (%)**	6.91 ± 1.30b	7.17 ± 1.58a	7.36 ± 1.62a	7.35 ± 1.46a	<0.001
**TG (mmol/l)**	1.82 ± 1.29	1.87 ± 1.44	1.98 ± 1.57	1.89 ± 1.43	0.376
**TC (mmol/l)**	5.03 ± 1.14a	5.16 ± 1.12a	5.17 ± 1.14a	5.21 ± 1.19a	0.039
**HDL-C (mmol/l)**	1.32 ± 0.43	1.35 ± 0.56	1.32 ± 0.51	1.33 ± 0.42	0.723
**LDL-C (mmol/l)**	2.93 ± 0.84	3.01 ± 0.93	3.06 ± 0.87	3.04 ± 0.94	0.080
**UAER**	9.5(6.4,22.8)	13.5(7.5,29.1)	11.1(7.0,27.4)	11.1(7.5,21.6)	0.074
**eGFR (ml/min)**	93.2(76.4,113.0)	91.4(71.9,114.6)	93.8(74.5,118.6)	94.5(74.8,117.4)	0.629

### The cumulative incidence rate of diabetes-related clinical events between 2014 and 2018

**Figure 2:**
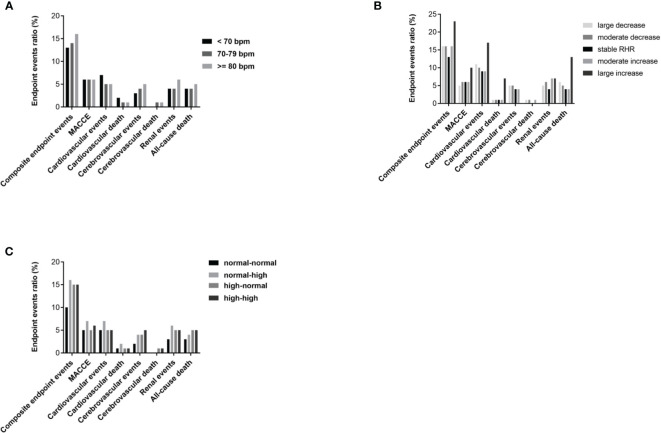
Cumulative incidence of diabetes-related clinical outcomes in T2DM patients from 2014 to 2018. **(A)** Comparison of prevalence of diabetes-related clinical outcomes between RHR<70 group, RHR 70-79 group and RHR>=80 group. **(B)** Comparison of prevalence of diabetes-related clinical outcomes between groups grouped according to the range of RHR change (large decrease group, moderate decrease group, stable RHR group, moderate increase group and large increase group). **(C)** Comparison of prevalence of diabetes-related clinical outcomes between groups grouped according to the trend of RHR change (normal-normal group, normal-high group, high-normal group and high-high group). P<0.05 was considered statistically significant.

The cumulative incidence rate of diabetes-related clinical events from 2014 to 2018 is shown in **Figure 1**. Out of 2,513 T2DM subjects, 106 cases suffered from all-cause death including 34 cardiac deaths, 14 cerebrovascular deaths and 58 deaths from other causes. There were 409 composite endpoint events, 143 MACCEs, 138 cardiovascular events, 98 cerebrovascular events and 117 renal events. There were no statistical differences in the incidence rate of various clinical events among the three groups (<70 group, 70-79 group and >=80 group), as shown in **Figure 2A**.

Grouped according to the range of RHR change and compared with the stable group, the large increase group, large decrease group and moderate decrease group had a significantly higher incidence of all-cause death, and the moderate increase group and moderate decrease group had a significantly higher incidence of renal events (both p< 0.001), as shown in **Figure 2B**. Grouped according to the trend of RHR change and compared with the normal-normal group, both the high-normal group and the high-high group had a significantly higher incidence of composite endpoint events and cerebrovascular events, and the normal-high group also had a significantly higher incidence of composite endpoint (both p< 0.001), as shown in **Figure 2C**.

### Cox proportional hazards models of the baseline RHR and the long-term RHR changes with future diabetes-related clinical outcomes

**Figure 3:**
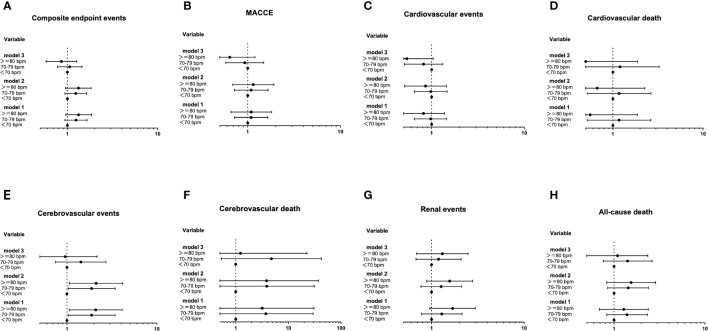
The Cox proportional hazards models of baseline RHR with future diabetes-related clinical outcomes **(A-H)**. Using the Entry method; crude and adjusted hazard ratios (HRs) with the 95% confidence intervals (CIs) given. Model 1 is an unadjusted analysis. Adjustment variables included age and gender in Model 2. In Model 3, the duration of diabetes, education level, smoking, waist circumference, concomitant disease history in baseline, ACEI/ARB use, β receptor blocker use, SBP, DBP, TC, HbA1c and eGFR were also considered other adjustment variables and were thus added to Model 2.

The Cox proportional hazards models of baseline RHR with future diabetes-related clinical outcomes were shown in **Figure 3**. In unadjusted analysis (Model 1), the risk of cerebrovascular events in 70-79 group and >=80 group were greater than that in <70 group, with HR (95% CI) of 1.88 (1.03-3.43), P=0.0039] and HR (95% CI): 2.09 (1.06-4.11), P=0.032], respectively. After modifying for age and gender (Model 2), the increase in risk remained statistically significant, as HR (95% CI): 1.88 (1.03-3.44), P=0.038] and HR (95% CI): 2.11 (1.07-4.18), P=0.030], respectively. However, after we further adjusted the duration of diabetes, education level, smoking, waist circumference, concomitant disease history in baseline, ACEI/ARB use, β receptor blocker use, SBP, DBP, TC, HbA1c and eGFR (Model 3), those differences became insignificant (P>0.05). For other clinical events, no significant difference was found among the three groups.

**Figure 4:**
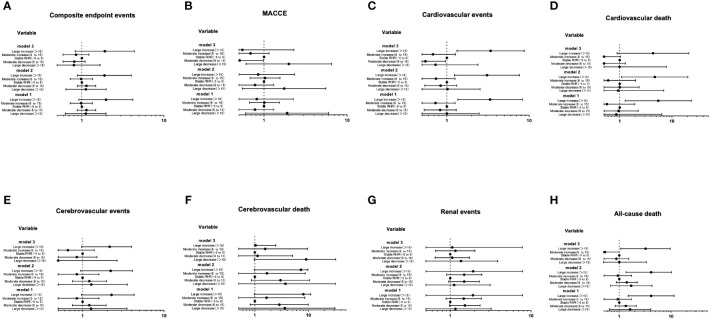
The Cox proportional hazards models of the range of RHR change with future clinical outcomes **(A-H)**. Using the Entry method; crude and adjusted hazard ratios (HRs) with the 95% confidence intervals (CIs) given. Model 1 is an unadjusted analysis. Adjustment variables included age and gender in Model 2. In Model 3, the duration of diabetes, education level, smoking, waist circumference, concomitant disease history in baseline, ACEI/ARB use, β receptor blocker use, SBP, DBP, TC, HbA1c, eGFR and the average RHR were also considered other adjustment variables and were thus added to Model 2.

The Cox proportional hazards models of the range of RHR change with future clinical outcomes were shown in **Figure 4**. Compared with the stable RHR group, after unadjustment (Model 1), adjustment for age and gender (Model 2) and further adjustment for some conventional risk factors and the average RHR (Model 3), the risk of cardiovascular events and all-cause death in the large increase group remained significantly high, with HR of 3.40 (1.33-8.71, P=0.010) and 3.22 (1.07-9.64, P=0.037), respectively; The risk of MACCE and all-cause death in the moderate decrease group remained significantly low, with HR of 0.51 (0.26-0.98, P=0.046) and 0.55 (0.31-0.96, P=0.037), respectively.

**Figure 5:**
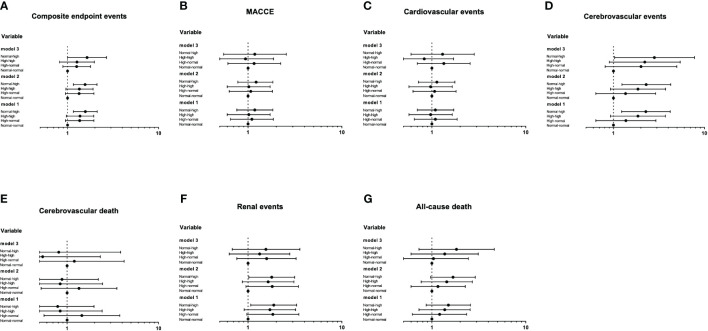
The Cox proportional hazards models of the trend of RHR with future clinical outcomes **(A-G)**. Using the Entry method; crude and adjusted hazard ratios (HRs) with the 95% confidence intervals (CIs) given. Model 1 is an unadjusted analysis. Adjustment variables included age and gender in Model 2. In Model 3, the duration of diabetes, education level, smoking, waist circumference, concomitant disease history in baseline, ACEI/ARB use, β receptor blocker use, SBP, DBP, TC, HbA1c, eGFR and the average RHR were also considered other adjustment variables and were thus added to Model 2.

The Cox proportional hazards models of the trend of RHR with future clinical outcomes were shown in **Figure 5**. Compared with the normal-normal group, the risk of composite endpoint events, cerebrovascular events and renal events were significantly increased in the normal-high group. After further adjusting for some traditional risk factors and the average RHR (Model 3), the risk of renal events was no longer significant (P>0.05), whereas the trend of composite endpoint events and cerebrovascular events in the normal-high group remained significant, with HR of 1.64 (1.00-2.68, P=0.047) and 2.82 (1.03-7.76, P=0.043), respectively.

## Discussion

This study finds that in the diabetic population, the RHR level at a time point is unable to predict the occurrence of long-term clinical events, while observing the pattern of RHR changes in a certain time can effectively predict the occurrence of long-term adverse events. Different patterns of RHR changes can predict different adverse outcomes. Moreover, these associations are robust and consistent in analyses adjusted for manifold traditional risk factors, as well as baseline beta-blocker use.

Numerous epidemiological studies have unveiled a vigorous correlation between elevated RHR and cardiovascular outcomes and mortality risks (all cause or cardiovascular). In the *post hoc* analysis of the ADVANCE study (a randomized clinical trial study of 12,500 T2DM patients), RHR was linked to cardiovascular events and all-cause mortality (14). In addition to confirming that the increase of RHR is an important predictor of cardiovascular disease, Miot et al. also found that RHR was a strong predictor for renal events when juxtaposed with CV events and when singled out from all other factors (15). These studies have all observed the correlation between baseline RHR levels and the occurrence of adverse events, while our study found no independent predictive value of baseline RHR levels for 10-year adverse clinical events. However, heart rate of people is not likely to remain steady throughout their lives. Therefore, whether temporal changes in RHR have predictive values is still poised to be an interesting and crucial issue.

Existing data indicate that an increase in RHR over time results in higher CV events and all-cause mortality in individuals with and without CVD (16–19). However, some studies were confined to certain sexes or health conditions, while others evaluated short-term RHR changes only (for example, over weeks or months). In our study, changes in RHR were monitored over a period of five years, which had the potential of measuring genuine alterations connected to physical fitness instead of short-term intraindividual variability. We adjusted for a wide range of potential confounders such as age, educational level, smoking, chronic conditions, drug usage and metabolic factors, which had yet to be done systematically in previous research. Results showed that compared with maintaining a stable RHR, the RHR significantly increased (over 15 bpm) within a certain period of time, cardiovascular events and all-cause mortality rate increased by 2.4 and 2.22 times, respectively. Compared with the RHR group who remained at normal level, the incidence of composite endpoint events and cerebrovascular events increased by 0.64 and 1.82 times in subjects with RHR ranging from normal to high level (from less than 70 bpm to greater than 70bpm) within half a decade.

Elevated RHR may have adverse effects on the body through different mechanisms. Firstly, high levels of RHR can heighten hemodynamic stress and shorten diastolic period, thus increasing mechanical load, shear stress, blood pressure and cardiac work. Such changes elevate oxygen consumption, leading to accelerated atherosclerosis and plaque rupture (20). Secondly, an increase in RHR has negative effects on the autonomic nervous system, tilting balance towards enhanced sympathetic nervous tension, which may cause life-threatening ventricular arrhythmias and sudden cardiac death (21). Thirdly, the main metabolic pathway of myocardial energy production in diabetes patients depends on nonesterified fatty acids. Compared with glucose oxidation, RHR entails a higher basal oxygen level (22). Furthermore, as shown in this study, apart from direct effects, increased heart rate is also relevant to various abnormal metabolic factors, such as abdominal obesity, elevated blood pressure, elevated TC and smoking. These factors themselves can have adverse effects on prognosis.

The shape of the associations between RHR and CVD morbidity and mortality across the full range of RHR has been reported as linear (23–25), sigmoid (26), ‘J’-shaped (27) or ‘U’-shaped (28), which results in the question whether a decreased RHR would bring clinical benefits. In our study, a decrease in RHR did not increase the risk of long-term adverse events in T2DM, while a moderate decrease in RHR (a decrease of 6-15bpm) could reduce the incidence of all-cause death and MACCE by 45% and 49%, respectively. Whether reducing RHR will have benefits for adverse outcomes in T2DM populations cannot be merely determined through cohort follow-up, and clinical trials are needed to clarify.

Our study has several limitations that must be recognized. Firstly, this study is not based on a random sampling survey of natural populations, so there could be some selection bias during the process of research. Secondly, given the observational nature of our study, we cannot completely rule out the possibility that some observed associations are caused by unmeasured confounding factors. Thirdly, we did not record any relevant content on physical exercises, which may have some impact on RHR. Fourthly, there is a lack of information on drugs (such as β receptor blockers) that may alter RHR during the second follow-up recording, which is a powerful potential confounding factor. Previous studies (17), however, have not pointed out the strong confounding effect of this drug. Fifthly, patients with a history of cardiovascular disease at baseline were not divided into subgroups for further analysis. Finally, observing the occurrence of major vascular events may take longer, and our study’s follow-up time is relatively short. Therefore, we need to continue our follow-up to observe the predictive value of temporal changes in RHR for adverse events in T2DM. However, we have no reasons to believe these would substantially bias the associations reported herein.

## Conclusion

To conclude, in T2DM population, temporal changes in RHR rather than a single point in time had certain predictive value for long-term clinical events in diabetes population. Compared to maintaining an appropriate and stable level of RHR, individuals with significantly elevated RHR over a given period of time had an increased risk of adverse events. While, a moderate decrease in RHR may have certain clinical benefits. Information about RHR and its time-related changes is not difficult to acquire and track, so monitoring RHR changes can help identify subgroups with high incidence of adverse events.

## Data availability statement

The raw data supporting the conclusions of this article will be made available by the authors, without undue reservation.

## Ethics statement

The studies involving humans were approved by the Ethics Committee of Beijing Tongren Hospital, Capital Medical University. The studies were conducted in accordance with the local legislation and institutional requirements. The participants provided their written informed consent to participate in this study.

## Author contributions

LG: Writing – review & editing, Writing – original draft, Methodology, Conceptualization. GW: Writing – review & editing, Supervision, Project administration. GW: Writing – original draft, Formal analysis, Data curation. QL: Writing – original draft, Investigation, Data curation. MQ: Writing – review & editing, Supervision, Project administration. FF: Writing – original draft, Investigation, Data curation. XC: Writing – original draft, Investigation, Data curation. YL: Writing – original draft, Investigation, Data curation. FS: Writing – original draft, Investigation, Data curation. XZ: Writing – original draft, Investigation, Data curation. HF: Writing – review & editing, Supervision, Methodology. SY: Writing – review & editing, Supervision, Resources, Project administration, Conceptualization.

## References

[1] Sarwar N Gao P Seshasai SR Gobin R Kaptoge S Di Angelantonio E. Diabetes mellitus, fasting blood glucose concentration, and risk of vascular disease: a collaborative meta-analysis of 102 prospective studies. Lancet. (2010) 375:2215–22. doi: 10.1016/s0140-6736(10)60484-9 PMC2904878 20609967

[2] Strath SJ Swartz AM Bassett DR Jr. O’Brien WL King GA Ainsworth BE. Evaluation of heart rate as a method for assessing moderate intensity physical activity. Med Sci Sports Exerc. (2000) 32:S465–70. doi: 10.1097/00005768-200009001-00005 10993416

[3] Zhang D Shen X Qi X. Resting heart rate and all-cause and cardiovascular mortality in the general population: a meta-analysis. Cmaj. (2016) 188:E53–e63. doi: 10.1503/cmaj.150535 26598376 PMC4754196

[4] Paul L Hastie CE Li WS Harrow C Muir S Connell JM. Resting heart rate pattern during follow-up and mortality in hypertensive patients. Hypertension. (2010) 55:567–74. doi: 10.1161/hypertensionaha.109.144808 20038750

[5] Sharashova E Wilsgaard T Mathiesen EB Løchen ML Njølstad I Brenn T. Resting heart rate predicts incident myocardial infarction, atrial fibrillation, ischaemic stroke and death in the general population: the Tromsø Study. J Epidemiol Community Health. (2016) 70:902–9. doi: 10.1136/jech-2015-206663 26951324

[6] Fox K Ford I Steg PG Tendera M Robertson M Ferrari R. Heart rate as a prognostic risk factor in patients with coronary artery disease and left-ventricular systolic dysfunction (BEAUTIFUL): a subgroup analysis of a randomised controlled trial. Lancet. (2008) 372:817–21. doi: 10.1016/S0140-6736(08)61171-X 18757091

[7] Böhm M Swedberg K Komajda M Borer JS Ford I Dubost-Brama A. Heart rate as a risk factor in chronic heart failure (SHIFT): the association between heart rate and outcomes in a randomised placebo-controlled trial. Lancet. (2010) 376:886–94. doi: 10.1016/s0140-6736(10)61259-7 20801495

[8] Ma YC Zuo L Chen JH Luo Q Yu XQ Li Y. Modified glomerular filtration rate estimating equation for Chinese patients with chronic kidney disease. J Am Soc Nephrol. (2006) 17:2937–44. doi: 10.1681/asn.2006040368 16988059

[9] Alberti KG Zimmet PZ. Definition, diagnosis and classification of diabetes mellitus and its complications. Part 1: diagnosis and classification of diabetes mellitus provisional report of a WHO consultation. Diabetes Med. (1998) 15:539–53. doi: 10.1002/(sici)1096-9136(199807)15:7<539::Aid-dia668>3.0.Co;2-s 9686693

[10] James PA Oparil S Carter BL Cushman WC Dennison-Himmelfarb C Handler J. 2014 evidence-based guideline for the management of high blood pressure in adults: report from the panel members appointed to the Eighth Joint National Committee (JNC 8). Jama. (2014) 311:507–20. doi: 10.1001/jama.2013.284427 24352797

[11] Kristal-Boneh E Silber H Harari G Froom P. The association of resting heart rate with cardiovascular, cancer and all-cause mortality. Eight year follow-up of 3527 male Israeli employees (the CORDIS Study). Eur Heart J. (2000) 21:116–24. doi: 10.1053/euhj.1999.1741 10637085

[12] Erikssen G Liestøl K Bjørnholt J Thaulow E Sandvik L Erikssen J. Changes in physical fitness and changes in mortality. Lancet. (1998) 352:759–62. doi: 10.1016/s0140-6736(98)02268-5 9737279

[13] Ho JE Bittner V Demicco DA Breazna A Deedwania PC Waters DD. Usefulness of heart rate at rest as a predictor of mortality, hospitalization for heart failure, myocardial infarction, and stroke in patients with stable coronary heart disease (Data from the Treating to New Targets [TNT] trial). Am J Cardiol. (2010) 105:905–11. doi: 10.1016/j.amjcard.2009.11.035 20346304

[14] Hillis GS Woodward M Rodgers A Chow CK Li Q Zoungas S. Resting heart rate and the risk of death and cardiovascular complications in patients with type 2 diabetes mellitus. Diabetologia. (2012) 55:1283–90. doi: 10.1007/s00125-012-2471-y PMC4170780 22286552

[15] Miot A Ragot S Hammi W Saulnier PJ Sosner P Piguel X. Prognostic value of resting heart rate on cardiovascular and renal outcomes in type 2 diabetic patients: a competing risk analysis in a prospective cohort. Diabetes Care. (2012) 35:2069–75. doi: 10.2337/dc11-2468 PMC3447829 22815300

[16] Seviiri M Lynch BM Hodge AM Yang Y Liew D English DR. Resting heart rate, temporal changes in resting heart rate, and overall and cause-specific mortality. Heart. (2018) 104:1076–85. doi: 10.1136/heartjnl-2017-312251 29269380

[17] Jouven X Empana JP Escolano S Buyck JF Tafflet M Desnos M. Relation of heart rate at rest and long-term (>20 years) death rate in initially healthy middle-aged men. Am J Cardiol. (2009) 103:279–83. doi: 10.1016/j.amjcard.2008.08.071 19121452

[18] Chen XJ Barywani SB Hansson PO Östgärd Thunström E Rosengren A Ergatoudes C. Impact of changes in heart rate with age on all-cause death and cardiovascular events in 50-year-old men from the general population. Open Heart. (2019) 6:e000856. doi: 10.1136/openhrt-2018-000856 31168369 PMC6519434

[19] Cui X Mandalenakis Z Thunström E Fu M Svärdsudd K Hansson PO. The impact of time-updated resting heart rate on cause-specific mortality in a random middle-aged male population: a lifetime follow-up. Clin Res Cardiol. (2021) 110:822–30. doi: 10.1007/s00392-020-01714-w PMC8166682 32696079

[20] Palatini P. Heart rate and the cardiometabolic risk. Curr Hypertens Rep. (2013) 15:253–9. doi: 10.1007/s11906-013-0342-7 23645136

[21] La Rovere MT Bersano C Gnemmi M Specchia G Schwartz PJ. Exercise-induced increase in baroreflex sensitivity predicts improved prognosis after myocardial infarction. Circulation. (2002) 106:945–9. doi: 10.1161/01.CIR.0000027565.12764.E1 12186798

[22] Boudina S Abel ED. Diabetic cardiomyopathy, causes and effects. Rev Endocr Metab Disord. (2010) 11:31–9. doi: 10.1007/s11154-010-9131-7 PMC2914514 20180026

[23] Kannel WB Wilson P Blair SN. Epidemiological assessment of the role of physical activity and fitness in development of cardiovascular disease. Am Heart J. (1985) 109:876–85. doi: 10.1016/0002-8703(85)90653-2 3885703

[24] Mensink GB Hoffmeister H. The relationship between resting heart rate and all-cause, cardiovascular and cancer mortality. Eur Heart J. (1997) 18:1404–10. doi: 10.1093/oxfordjournals.eurheartj.a015465 9458445

[25] Seccareccia F Pannozzo F Dima F Minoprio A Menditto A Lo Noce C. Heart rate as a predictor of mortality: the MATISS project. Am J Public Health. (2001) 91:1258–63. doi: 10.2105/ajph.91.8.1258 PMC1446757 11499115

[26] Palatini P Casiglia E Julius S Pessina AC. High heart rate: a risk factor for cardiovascular death in elderly men. Arch Intern Med. (1999) 159:585–92. doi: 10.1001/archinte.159.6.585 10090115

[27] Kuzuya M Enoki H Iwata M Hasegawa J Hirakawa Y. J-shaped relationship between resting pulse rate and all-cause mortality in community-dwelling older people with disabilities. J Am Geriatr Soc. (2008) 56:367–8. doi: 10.1111/j.1532-5415.2007.01512.x 18251825

[28] Dyer AR Persky V Stamler J Paul O Shekelle RB Berkson DM. Heart rate as a prognostic factor for coronary heart disease and mortality: findings in three Chicago epidemiologic studies. Am J Epidemiol. (1980) 112:736–49. doi: 10.1093/oxfordjournals.aje.a113046 7457467

